# Interactive effects of telomere length, mtDNA abundance, and epigenetic age on Alzheimer’s disease endophenotypes

**DOI:** 10.1002/alz70861_107957

**Published:** 2025-12-23

**Authors:** Shea J Andrews, Brendan A Mitchell, Tong Tong, Luke W. Bonham, Alan E. Renton, Xiaoling Zhang, Marina Sirota, Duygu Tosun, Kristine Yaffe

**Affiliations:** ^1^ Department of Psychiatry and Behavioral Sciences, University of California ‐ San Francisco, San Francisco, CA USA; ^2^ University of California San Francisco, San Francisco, CA USA; ^3^ Boston University, Boston, MA USA; ^4^ Memory and Aging Center, Department of Neurology, Weill Institute for Neurosciences, University of California, San Francisco, San Francisco, CA USA; ^5^ Icahn School of Medicine at Mount Sinai, New York, NY USA; ^6^ Bakar Computational Health Sciences Institute, University of California, San Francisco, San Francisco, CA USA; ^7^ San Francisco Veterans Affairs Medical Center, San Francisco, CA USA; ^8^ University of California, San Francisco, San Francisco, CA USA

## Abstract

**Background:**

Biological age, reflecting the cumulative molecular and cellular damage such as telomere attrition, epigenetic alterations and mitochondrial dysfunction, may better capture age‐related decline and Alzheimer’s disease (AD) risk than chronological age. Yet, the independent and interactive contributions of biological age markers for telomere length (TL), epigenetic DNA methylation age (DNAm age), and mitochondrial DNA copy number (mtDNAcn) to AD pathogenesis remain unclear.

**Methods:**

We estimated blood‐derived TL via qPCR, DNAm age using the CausAge clock from DNAm array, and mtDNAcn from whole genome sequencing in 640 participants from the Alzheimer’s Disease Neuroimaging Initiative (ADNI; Age: 74.95±7.62, Female: 44.2%, cognitively unimpaired: 33.8%, mild cognitive impairment: 51.6%, AD: 14.7%). Linear mixed‐effects models examined the associations and interactions of these markers with a global cognitive composite score, while linear regression tested associations with baseline cerebrospinal fluid (CSF) Aβ_42_, total‐tau, and phosphorylated tau as well as with cortical thickness and gray matter volume at last visit. Models adjusted for baseline age, sex, clinical dementia rating scale, *APOE*, blood cell composition, and outcome‐specific covariates (education, scanner, and intracranial volume).

**Results:**

Individually, TL, DNAm age, and mtDNAcn were not associated with cognition, CSF biomarkers, or neuroimaging outcomes. However, mtDNAcn moderated the association of both TL and DNAm age with baseline cognitive performance such that among individuals with shorter telomeres (β [SE] = 0.033 [0.014], *p* =0.02) or elevated DNAm age (‐0.049 [0.026], *p* =0.065), higher mtDNAcn was associated with poorer cognition. Additionally, there was a synergistic interaction between CausAge and TL on CSF Aβ_42_ (‐4.9e‐05 [1.6e‐05], *p* =0.002), Aβ_42_ levels were lower in individuals with higher CausAge and shorter telomeres.

**Conclusion:**

These findings suggest that increased mtDNAcn may act as a compensatory response to accelerated epigenetic aging and telomere attrition; and that epigenetic age and telomere attrition are associated with greater Ab deposition. Our results underscore the importance of evaluating the interplay among multiple biological aging markers when investigating AD pathogenesis.

